# On Medical Reform

**Published:** 1806-11

**Authors:** 


( To be Continued. )

---

### *To the Editors of the Medical and Physical Journal.*

Gentlemen,

I Presume the generality of professional men, will admit that a reformation in medicine is of late become much wanted in this kingdom, and I sincerely wish to see the science, in all its various departments, divested of innovation and empiricism. I have paid some little attention to the numerous letters on the subject of Medical Reform, which have appeared in some periodical works; together with the circular letter exhibited to the public by the projector of this scheme, amongst which, when taken collectively, it is evident many of the authors of these attempts at reform, appear to have undertaken the subject on too narrow a scale, and thereby each practitioner seems, through this medium, to be endeavouring from selfish motives, to redress his own grievances, or those of his colleagues, without first dispassionately weighing the disadvantages resulting therefrom, to the profession and community at large. It would appear from these written documents, that the burden and weight of grievances lay with the physicians; but when fairly investigated, it will be found, in reality, to rest amongst the surgeons and apothecaries; and that the physicians, generally considered, are the sole *cause* of these depredations in practice. This is easily proved. First, by the circumstance of the physicians encouraging their prescriptions being prepared by druggists. Secondly, by their infringements on the province of the surgeon, in performing even the most trivial operations. And, thirdly, by their encroachment on the accoucheur, by attending cases of parturition; in short, in this part of the kingdom, instances of this opprobrious kind are become so notorious, that the surgeon, apothecary, and man-midwife, can scarcely obtain a decent livelihood; nay, to the physician's shame, it is not uncommon to observe him undertake the whole departments of the profession; and my astonishment is further heightened, to learn that the worthy Chairman of the Medical Benevolent Society of Lincolnshire is one amongst the number of physicians in Great Britain, who has himself long encroached on the Chirurgical Department.* How absurd then must it appear to the Legislature, to hear a body of men murmuring under a load of grievances, and at the same time, duplicitly petitioning for the redress of abuses, which they are at this moment encouraging, either directly or indirectly. It is evident that the physicians have long attempted to arrogate all the merit, interest and practice of the profession to themselves and the druggists, by which detestible and lucriferous conduct, the life of the patient becomes frequently endangered, and the apothecary deprived of his just privilege and reward.

It is not my intention at present in this letter to depict to the reader, every professional infringement, nor estimate the evils which I have so frequently witnessed, both amongst those who are called regular and irregular practitioners. The importance of the healing art, in all its different branches, is universally acknowledged of the greatest moment to the welfare of the nation; hence may be urged the necessity of its being established on the best of principles, and entrusted to those only who are capacitated to undertake and fulfil the office with honour to the profession and fidelity to the public. It is I believe an incontrovertible fact, and will be universally admitted, even by professional men, that there is more illiberality, and a greater want of candour, in this branch of science, than any

* It is a rule with the College of Edinburgh, when a degree is conferred on a student, that each graduate take an oath that he will practice his profession in a just and honourable way; how far the promoters of this crude reform, have acted consistent with this solemn appeal to the duty, is sufficiently known amongst their neighbouring practitioners.

any other; and the fountain from whence this noxious evil springs, will on enquiry be found to arise chiefly from professional jealousy, and motives of self-interest. It appears then essentially necessary, first, that these weeds of corruption should be exterminated from among the faculty, before any attempts be made to invoke from the legislature, a reform in medicine. I presume no further apology will be necessary for these animadversions, than a sincere wish to see the science of medicine restored to its genuine and pristine state; and it is incumbent on every practitioner to endeavour, to the utmost of his power, to abolish this corrupt degeneracy, which has so long degraded the healing art. And to effectuate so desirable a purpose, and re-establish the science on a better foundation, I conceive there requires little more to be done, than for the College of Physicians and Corporation of Surgeons of London, to exert their power in future to the utmost, in a coercive way on the faculty, when I am persuaded, one great step will be attained towards a radical reform; in the mean time, I cannot help commiserating the Members of the Medical Benevolent Society of Lincolnshire, who seem anxiously engaged in usurping to themselves, a power similar to that of the Royal College of Physicians in London; I trust, however, that the Members of the Benevolent Society, when taken collectively, are governed by a superior motive, than that of simply obtaining popularity; nevertheless, I am disposed to believe, that if a general appeal had been made from the faculty at large to the College of Physicians and Corporation of Surgeons, and their efforts become united on this important occasion, more good would have resulted from their joint endeavours, than what appears likely to be obtained by this premature and discordant step. Empiricism has long been carried on in this town and neighbourhood, to very destructive lengths, down from the clergy* to the meanest mechanic; and in some parts of this county, we frequently see the physician vend medicine, the surgeon apothecary, and man-midwife, employed (from mere necessity)

* It is very common in this part of the county, for the clergy of every denomination, to prescribe medicines for the sick, and direct their patients to get them compounded at the druggists; and I am sorry to add that some have even violated the dignity of their functions, by condescending to promulgate the absurd doctrine of Perkins; for I have been consulted in cases where these scenes of infatuation, by means of the metallic tractors, had by them been conducted with the greatest parade and solemnity.

sity) in trade or husbandry; the druggist occupied in farriery, or a dealer in groceries, and vice versa. In short, these different branches of medical science, now appear so interwoven with each other, and are in that state of degeneracy, that it is scarcely possible to point out that shade of distinction, which was once thought so essentially necessary, to form a regular and scientific practitioner. It may be thought unnecessary to say where, or by what means, some of these men of letters have procured their diplomas; but I will hazard a conjecture, that if the College of Physicians were to exert their legal authority of enforcing a general examination, so far as their prerogative extends, it is more than probable many would be found wanting in practical knowledge, when tried by this criterion.

"There is not, (says Mr. Good, in his History of Medicine) perhaps a single druggist in the whole kingdom, who compounds his different preparations in all respects, consistently with the College Dispensatory; but the druggists at Manchester, appear to excel all others in such nefarious ingenuity;" to which work I will refer the reader, wherein he will find a collection of these fatal succedaneums, which manifestly prove the evil tendency of the physicians entrusting their prescriptions to be prepared by these ignorant druggists. I am acquainted with a physician in one of the largest towns in this county, who lately presented himself as a candidate before the College of Physicians in London, and who has since pompously given publicity to his competency, of practising as physician in future, by an advertisement in several provincial newspapers. This learned Doctor did once, most assuredly, prevail on a young surgeon (a pupil of his) to relinquish the idea of his own profession for that of a druggist, from what mercenary views I do not undertake to explain; but the scheme was put into execution, and the druggist has been heard to aver that the Doctor made a point of sending and recommending his prescriptions, to be prepared at his shop, without making any enquiry respecting the quality of the drugs, or the competency of those by whom they were compounded. Whatever plea the physician may have, for thus subverting the long established order in these different departments of science, I cannot pretend to say, for I do not find them governed by any such exclusive prerogative, either ancient or modern, nor are there any accounts of druggists to be found in any Author, before the close of the year 1553; but the professions of the apothecary and surgeon are of very remote date, and even even claim free privilege, and support a priority with that of the physician; and if he will revert to the history and progress of medicine, that research will easily convince the enquirer, that the art of medical surgery is nearly coeval with time itself;* from this source will also be derived, the period in which the division of these branches of learning and practice took place. To what circumstance this distinction of surgeon, apothecary, and physician can be attributed, I will not in this place attempt to set forth; but there can scarcely be a doubt that many important advantages were derived to society, and the dignity and rank of the profession heightened thereby. This æra took place, if I mistake not, within a few centuries after the time of Hippocrates, before which period the physician was occasionally, and, perhaps, in some cases from necessity and the state of society, engaged in the chirurgical and pharmaceutical departments; and the surgeon likewise exhibited internal medicines, even in diseases which had no affinity with his own distinct art. Hence it appears from history, that the science of medicine, like most other arts, has, through a succession of ages, undergone a variety of changes and improvements; and such is the opinion of the most enlightened of the faculty of the present day, that each practitioner, whether physician or surgeon, should now assume no other rank or privilege, than that of his own distinct order. On this subject, the late Dr. Percival thus expressly delivers his opinion, in his Medical Ethics, "In large and opulent towns, (says he) the distinction between the province of physic and surgery should be steadily maintained; this distinction is sanctioned both by reason and experience. It is founded on the nature and objects of the two professions, on the education and acquirements, requisite for their most beneficial and honourable exercise: and tends to promote the complete cultivation and advancement of each."

I am, &c.

A REGULAR PRACTITIONER.

*Lancashire, Aug.* 27, 1806.

( To be continued. )

To

* We read of the healing of wounds in Scripture, see Deutr. Chap. 32, verse 39, even 600 years before the time of Homer; and near 1000 before Hippocrates.

